# Inappropriate medication use among the elderly: a systematic review of administrative databases

**DOI:** 10.1186/1471-2318-11-79

**Published:** 2011-11-30

**Authors:** Lusiele Guaraldo, Fabíola G Cano, Glauciene S Damasceno, Suely Rozenfeld

**Affiliations:** 1 Escola Nacional de Saúde Pública Sérgio Arouca-Fundação Oswaldo Cruz, Rio de Janeiro, Brazil; 2 Instituto de Pesquisa Clínica Evandro Chagas-Fundação Oswaldo Cruz, Rio de Janeiro, Brazil; 3 Universidade Estadual do Oeste do Paraná, Cascavel, Brazil

## Abstract

**Background:**

Inappropriate medication use (IMU) by elderly people is a public health problem associated with adverse effects on health. There are a number of methods for identifying IMU, some involving clinical judgment and others, consensually generated lists of drugs to be avoided. This review aims to describe studies that used information from insurance company and social security administrative databases to assess IMU among community-dwelling elderly and to present the risk factors most often associated with IMU.

**Methods:**

The paper search was conducted in Medline and Embase, using descriptors combined with free terms in the title or abstract. The limits applied were: publication date from January 1990 to June 2010, species (human) and publication type (excluding editorials, letters and reviews). Excluded were: case studies; studies in hospitals, nursing homes, or hospital emergency departments; studies of specific drugs or groups of drugs; studies exclusively of subgroups of ill, frail elderly or rural populations. Additional studies were identified from reference lists. Data were selected and extracted after independent reading by two of the authors, with disagreements resolved by a third author. The primary outcome assessed was prevalence of IMU, defined as the proportion of elderly who received at least one inappropriate medication.

**Results:**

Of the 628 studies, 19 met the inclusion criteria, 78.9% of them conducted in the USA. All papers included used explicit criteria of inappropriateness, most commonly Beers criteria (73.7%) in their three versions (1991, 1997 and 2002). Other methods used included Zhan, which is derived from on Beers criteria and was applied in 21% of the papers selected. The study found that prevalence of IMU ranged from 11.5% to 62.5%. Only 68.4% of the studies included examined inappropriate use-related factors, the most important being female sex, advanced age and larger number of drugs.

**Conclusions:**

The results show that the prevalence of IMU among community-dwelling elderly is high and depends partly on the method used to evaluate improper use. Besides the diversity of methods, other factors, such as patient sex, age and number of drugs used concurrently, appear to have influenced the estimates of IMU.

## Background

The elderly are the segment of society most exposed to medication. Studies in developed countries show that consumption of medication increases with age and that many elderly use at least three prescribed drugs concurrently [1,2]. In developing countries, the proportion of elderly using at least one medication daily ranges from 85 to 90% [3-6]. However, physiological and physiopathological peculiarities in this age group lead to differences in the pharmacokinetics and pharmacodynamics of the medications administered, making the prescription process complex and often inappropriate [7,8]. Studies show that elderly patients can present alterations in practically all pharmacokinetic processes (absorption, first-pass metabolism, bioavailability, distribution, protein building, renal and hepatic clearance). These alterations can lead to lower effectiveness of some drugs, such as enalapril, which is a pro-drug, and its bioavailability can be affected by reduction of the first-step metabolism [7]. On the other hand, they can also contribute to increasing risk of adverse drug reactions; for example, drugs with high hepatic-extraction ratios, such as the nitrates, barbiturates, lidocaine and propranolol, may have reduced hepatic metabolism in older adults [9].

Today, inappropriate prescribing for the elderly is considered a major public health problem, given its association with morbidity and mortality and in view of health service costs resulting from adverse reactions [10,11].

Various strategies have been developed to identify inappropriate prescription patterns. Methods are based on implicit criteria, involving clinical judgment grounded in reviews of the medical literature (Medication Appropriateness Index, for example [12]), and explicit criteria, based on consensually-generated lists of drugs to be avoided. One of the most used is the Beers method created in 1991 and updated in 1997 and 2002 [13-15].

The review by Jano & Aparasu [10] shows that, on the Beers criteria, use of inappropriate medication is associated with adverse effects on health, especially hospitalizations, among elderly residing in the community. Concomitant use of several medications (polypharmacy) is also related to adverse reactions, morbidity and mortality [16].

Various methods have been widely used in several countries to identify inappropriate prescription patterns and their effects on the health of the elderly, and to foster improved therapeutic practice [11,13]. Studies show that providing information of inappropriate medication use to health authorities can help improve pharmacotherapy among the elderly by providing input to regulatory action with a view to reducing inappropriate prescribing [17].

The aims of this review are to identify and describe studies that used information from insurance company and social security administrative databases to assess inappropriate medication use among community-dwelling elderly (60 years and older) and to present the risk factors most often associated with inappropriate medication use.

## Methods

### Search

The paper search was conducted on the Medline electronic database, using the PubMed interface. The MeSH descriptors used were "aged" not "frail elderly" combined with "drug therapy", "drug utilization", "pharmaceutical preparations", "drug interactions" and with the free terms "inappropriate drug", "inappropriate drugs", "inappropriate medication", "inappropriate medications", "inappropriate medicines", "inappropriate prescribing", "inappropriate prescription", "inappropriate prescriptions", "inadequate medication", "suboptimal therapy", "suboptimal prescribing" in the paper title or abstract. The search limits were: publication date from January 1990 to June 2010, species (human), and publication type (excluding editorials, letters and reviews). The search strategy was also performed in the Embase database (the complete search strategy is presented in Additional file 1).

### Selection

Papers were selected by two authors independently, and reviewed by a third author, according to the stages described below.

After reading the titles returned by the search, we excluded the following: case studies; studies in hospitals, nursing homes, or hospital emergency departments; studies of specific drugs or groups of drugs; and studies exclusively of subgroups of ill, frail elderly or rural populations. These same criteria were applied to the abstracts of the publications selected. Also excluded were guidelines and studies which offer no inappropriateness frequency estimates, as well as those without abstracts. The articles were selected with no language restriction.

On reading the abstracts it was possible, from the nature of the data, to identify two groups: studies of primary data sources and studies of secondary data sources. By reading the Methods section of each study they could be classified by the nature of the data used. This paper examines the studies of secondary data sources, i.e. insurance company and social security administrative databases developed primarily for purposes other than evaluating medication use. That choice was made in view of their representativeness and of the power to detect differences, because they contain records on large numbers of people.

The same inclusion and exclusion criteria were applied in order to select studies retrieved by manual search in the bibliographic references of the selected articles.

### Reading and data extraction

Each paper was examined for: population studied, inappropriateness criterion (Beers, Drug Utilization Review; Zhan; McLeod; Medication Appropriateness Index, and others), measures of frequency of inappropriate use (proportion of elderly), description of inappropriate medications (drugs or classes of drugs), and factors associated with improper use. The exclusion criteria mentioned above were applied to the full texts.

The data extraction form and the corresponding instruction manual for completing it were tested initially with five articles and subsequently subjected to minor adjustments, such as including new data record fields or changing format to accommodate information recording better. It comprised seven sections, which can be summarized as follows: identification of the article; description of the study source data base (type; country; scope); study population (individuals/visits/prescription); characterization of the participants (age; sex; schooling; income; co-morbidity); measures of inappropriate use frequency (proportion of elderly); inappropriateness criteria used (Beers, 1991; Beers, 1997; Beers, 2002; Drug Utilization Review; Zhan; McLeod; Medication Appropriateness Index, and others); medications (used; inappropriate by drugs/classes of drugs); associated factors (odds ratio; confidence intervals, p-value). The form is available from the authors.

During reading of the complete texts, data quality was also evaluated for inclusion in the review. Although the Strobe Initiative [18] is not a tool for evaluating study quality, some points from it were considered here for that purpose, especially as regards the Methods section, as follows: (1) Setting: describe the setting, locations, and relevant dates, including recruitment, exposure, follow-up, and data collection periods; (2) Participants: give the eligibility criteria, and the sources and methods of selection of participants; (3) Data search: give sources of data and details of methods of assessment (measurement). These items had to be present in an article in order for it to be included in the review.

A number of terms designating inappropriate medication use were encountered: drugs to be avoided in the elderly, inappropriate drug use, potentially inappropriate medication, potentially inappropriate prescribing in the elderly, and potentially inappropriate prescribing. In this review all these terms were expressed as IMU (inappropriate medication use).

### Analysis

Epidata was used for data input and analysis. A description of the studies is given as regards country and sample characteristics, inappropriateness criteria used in each article, and prevalence of IMU defined as the proportion of elderly who received at least one inappropriate medication. The factors associated with inappropriate use are also shown. Proportions were extracted to measure frequencies relating to the variables country, type of measurement of IMU used, and drugs/therapeutic classes most identified as inappropriate.

## Results

**Figure 1:**
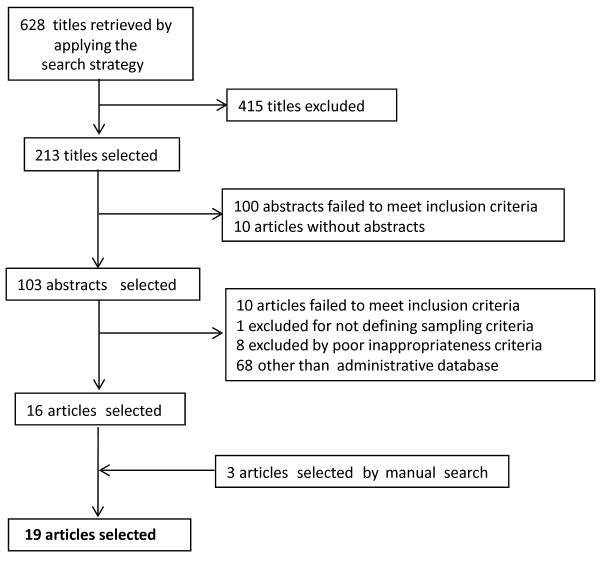
**Flowchart of systematic article search and selection for inappropriate medication use among elderly**.

The search strategy returned 628 papers (Medline and Embase); the exclusions at each stage are shown in Figure 1.

As regards the quality of the articles, we excluded one article for not defining sampling criteria and another 8 whose criteria of inappropriateness differed widely from previously validated criteria (such as Beers [13], Zhan [19] or McLeod [20]), i.e., they used criteria not specific to the elderly (2), or made extensive adaptations to drugs lists, resulting in distortion of validated criteria (4), or focused on overall quality of patient care (2).

After application of the inclusion and exclusion criteria, 19 studies were selected for analysis. In all these the population was studied retrospectively.

It was noted that, over the period reviewed (1990 to 2010), the number of papers published on IMU has increased steadily in more recent years, and more than doubled in the past five. Table 1 describes the main characteristics of the papers included in the review. They were produced in 5 different countries of North America, Europe, Oceania and Asia, 78.9% (15/19) of them in the USA.

**Table 1:** Prevalence* of Inappropriate Medication Use (IMU), characteristics of studies (1990-2010)

Reference/Year	Country/Cohort**	Population characteristics Sample(N); Sex (% Males); Age (years)	**Criteria**^#^	Prevalence
Lai et al., 2009 [21]	Taiwan/NHIRD	N = 2 133 864 elderly Age: ≥ 65	Beers 2002 (excluded drug-disease interaction, short-acting bzd, medications not marketed in Taiwan, not reimbursed by NHI and classified as second-degree controlled substances)	62.5%

Fick et al., 2008 [35]	USA/Southeast Managed Care Organization	N = 16 877 elderly Males:39% *** Age: ≥ 65/mean = 73.3(SD = 6.47)	Beers 2002 (do not use, excluded oxybutinin, dose)	40.7%**^§^**

Pugh et al., 2008 [26]	USA/VA	N = 850 154 elderly Males: 98% Age: ≥ 65	Zhan	26.2%

Bierman et al., 2007 [27]	USA/VA	N = 965 756 elderly Males: 98%*** Age: ≥ 65 years	Zhan	23% (men); 26.7% (women)

Roughead et al., 2007 [23]	Australia/VA	N = 192 363 elderly Males: 52.6% Age: ≥ 70/mean = 81.7(SD = 4.8)	Beers 2002/McLeod (do not use)	21.2%**^§^**

Barnett et al., 2006 [28]	USA/VA	N = 123 633 elderly Males:97.3% Age: ≥ 65/mean = 74.7(SD = 5.8)	Zhan	21.3%**^§§^**

Maio et al., 2006 [22]	Italy/Emilia Romagna outpatient prescriptions claims database	N = 849 425 elderly Males: 41.1%*** Age: ≥ 65/mean = 75.6(SD = 7.5)	Beers 2002 (do not use; excluded medications not marketed in Italy or not reimbursed by the Italian National Formulary)	18%

Pugh et al., 2006 [30]	USA/NPCD/VA/Large Health Survey of Veterans	N = 1 096 361 elderly Males: 98%*** Age: ≥ 65	HEDIS 2006	19.6%

Zuckerman et al., 2006 [34]	USA/MarketScan Medicare Supplemental and Coordination of Benefits	N = 487 383 elderly Males: 55.5% Age: ≥ 65/mean = 73.8(SD = nr)	Beers 2002	41.9%

Pugh et al., 2005 [24]	USA/VA	N = 1 265 434 elderly Males: 98% Age: ≥ 65/mean = 73.5(SD = 5.6)	Beers 1997 (dose)/Zhan	23%

Rigler et al., 2005 [36]	USA/Kansas Medicaid beneficiaries	N = 1 163 elderly Males: 30.5% Age: ≥ 60	Beers 1997 (do not use)	21%

Simon et al., 2005 [29]	USA/10 HMOs	N = 157 517 elderly Males: 43.5% Age: ≥ 65	Zhan	28.8% (95% CI 28.6-29.1) **^§§^**

Curtis et al., 2004 [37]	USA/Advanced PCS	N = 765 423 elderly Males: 41.7%*** Age: ≥ 65/mean = 73.7(SD = 6.5)	Beers 1997 (do not use)	21.2%

Howard et al., 2004 [25]	Canada/OCB/RPDB	N = 777 elderly Males: 37.5% Age: ≥ 65/mean = 74.1(SD = 6.0)	Beers 1991/Beers 1997 (included bzd with > 30 days supply and > 1 bzd or NSAID simultaneously)	16.3%

Rigler et al., 2004 [39]	USA/Kansas Medicaid beneficiaries	N = 1 163 elderly Males: 30.5% Age: ≥ 65	Beers 1997 (do not use)	21%

Fick et al., 2001 [33]	USA/Southeastern HMO	N = 2 336 elderly Males: 40.2%*** Age: ≥ 65	Beers 1997(do not use)	24.2%

Mott & Meek, 2000 [38]	USA/Database of ambulatory pharmacies of a Midwestern state	N = 1 185 elderly Males:35.7%*** Age: range 65-97/mean = 74.9(SD = 7.06)	Beers 1997 (do not use)	14.3%

Piecoro et al., 2000 [31]	USA/Kentucky Medicaid Recipients	N = 44 259 elderly Age: ≥ 65	Beers 1997 (do not use, excluded antihistamines)	24.4%

Futterman et al., 1997 [32]	USA/HMO Medicare plan/PBM	N = 10 076 elderly Age: ≥ 65	Beers 1991	11.53% (1994); 12.8% (1993)

* in the preceding year

** Advanced PCS = outpatient prescription claims database of national pharmaceutical benefit manager; HMO = Health Maintenance Organizations; NHI = National Health Insurance program; NHIRD = National Health Insurance Research Database - year 2004; NPCD = National Patient Care Database; OCB = Ontario Drug Benefit Plan; PBM = Pharmaceuticals benefit manager; RPDB = Registered Persons Database; VA = Veterans Affairs administrative and pharmacy database.

*** Recovered values; nr = not reported

**^# ^**data in brackets refer to the subtype or modification of criteria used in the study: "do not use" refers to drugs that should be avoided in any circumstances, "dose" refers to drug doses that should not be exceeded and "drug-disease interactions" refers to drugs to avoid in patients with specific conditions; bzd = benzodiazepines; HEDIS = Healthcare Effectiveness Data and Information measures.

**^§ ^**prevalence in the preceding 6 months; **^§§ ^**prevalence in the preceding 18 months

Thirty seven percent (7/19) of the studies were based on nationwide administrative databases. They all presented data on population size, expressed as numbers of individuals over 65 years old. One study (1/19) included only elderly over 70 years old, and one study (1/19) included elderly over 60 years old. Information on sex was included in 84.2% (16/19) of the studies. Study sample sizes ranged from 777 to 2 133 864 elderly.

All papers used explicit criteria of inappropriateness and 73.7% (14/19) used one of the three versions of Beers criteria (1991, 1997 and 2002). However, 85.7% (12/14) adapted the criteria to restrict them to inappropriate drugs regardless of dosage or specific clinical conditions or even to drugs available in the country of the study [21,22]. About 16.0% (3/19) used more than one criterion in order to evaluate combined inappropriate medication use [23-25]. Other methods used included Zhan and HEDIS (derived from Beers criteria), which were applied in 21% (4/19) [26-29] and 5.3% (1/19) [30] of the studies, respectively.

There was clear variation in estimates of IMU prevalence. Among estimates generated by applying several criteria, prevalence of IMU ranged from 11.5% to 62.5% (Table 1).

As regards the drugs/therapeutic classes most identified as inappropriate, 36.8% (7/19) of the studies [22,27-29,31-33] described the inappropriate medication as individual drugs, two reported them as therapeutic classes [21,34]; nine presented rankings of classes and individual drugs [23-25,30,35-39], and one did not describe the inappropriate medications [26].

Of the studies that identified the inappropriate medications as individual drugs [22,27-29,31-33], 85.7% (6/7) mentioned amitriptyline; 85.7% (6/7), propoxyphene; and 51.1% (4/7) cyclobenzaprine, among the five inappropriate drugs most used.

Factors associated with IMU were addressed in 68.4% (13/19) of the studies. About 15.8% (3/19) used univariate analyses [29,32,38] and 52.6% (10/19) multivariate analyses. The factors most often associated with IMU include: female sex, age and number of drugs prescribed or dispensed (Table 2).

**Table 2:** Factors associated with inappropriate medication use (IMU) in multivariate analysis*, articles published between 1990 and 2010

Reference/Year	Sex	Age	No. of medications
Lai et al., 2009 [21]	Male (ref. Female) 0.98 (0.98-0.98)	Age (ref. 65-69) 70-74: 0.99 (0.99-0.99) 75-79: 1.00 (0.99-1.00) ≥ 80: 1.02 (1.02-1.02)	No. of medications 4-6: 2.70 (2.69-2.70) ≥ 7: 4.53(4.52-4.54)

Pugh et al.,2008 [26]	Female (ref. Male) 1.25 (1.19-1.32)	Age (ref. ≥ 85) 65-74: 1.16 (1.11-1.22) 75-84: 1.08 (1.04-1.12)	Unique drugs (ref. ≥ 10) 1-3: 0.10(0.10-0.11) 4-6: 0.25 (0.24-0.26) 7-9: 0.46 (0.45-0.47)

Bierman et al.,2007 [27]	Female (ref. Male) 1.23 (1.18-1.27)	Age (ref. ≥ 85) 65-74: 1.11 (1.07-1.14)**^m^** 75-84: 1.05 (1.02-1.09)**^m^**	No. of medications 1.17 (1.17-1.17)**^m^** 1.18 (1.17-1.19) **^w^**

Maio et al.,2006 [22]	Female (ref. Male) 0.92 (0.91-0.93)	Age (ref. 65-74) 75-84: 1.11 (1.09-1.12) ≥ 85: 1.18 (1.16-1.20)	No. of drugs prescribed (ref. 1-3) 4-6: 2.37 (2.31-2.42) 7-9: 3.91 (3.82-4.01) ≥ 10: 7.33 (7.15-7.51)

Pugh et al.,2006 [30]	-	Age (ref. ≥ 85) 65-69: 1.30 (1.20-1.30)**^m^** 1.30(1.10-1.60)**^w^** 70-84: 1.10 (1.10-1.10)^m^ 1.00(0.90-1.20)^w^	Unique medications (ref. 1-3) 4-6: 2.20(2.10-2.20)**^m^** 2.30(1.90-2.70)**^w^** 7-9: 3.80(3.80-3.90)**^m^** 4.30(3.70-5.10)**^w^** ≥ 10: 8.20(8.00-8.40)**^m^** 9.60(8.20-11.20)**^w^**

Howard et al., 2004 [25]	Female (ref. Male) 1.60 (1.00-2.40)	-	-

Rigler et al, 2004 [39]	Female **	-	No. of prescriptions per month **

* Values are OR adjusted (95% CI). Factors for adjustment: Lai (2009): physician characteristics (sex, age, and specialty), and visit characteristics; Pugh (2008): race, mental comorbidities, geriatric care; Bierman (2007): race/ethnicity, psychiatric comorbidity, health care utilization, visits in primary care; Maio (2006): geographic location; income; chronic condition drug group; Pugh (2006): race/ethnicity; psychiatric comorbidity, serious mental illness or other mental health diagnoses, outpatient visits; Howard (2004): education, self-reported health; number of conditions. Rigler (2004): age, race.

**^m ^**value in men; **^w ^**value in women

** Female sex and number of prescription per month were associated with higher levels of inappropriate medication use, p ≤ 0.01.

## Discussion

Our results suggest that prevalence of IMU is high among community-dwelling elderly, and that this use is associated with the female sex, advanced age and the number of drugs prescribed.

Studies clearly varied widely in their estimates of prevalence of inappropriate medication use by the elderly. Among estimates generated by applying several criteria, prevalence of IMU ranged from 11.5% to 62.5%. This variability may result from a number of factors, among them the diversity of inappropriateness criteria.

Most of the studies used classic explicit criteria, such as Beers. The Beers criteria, developed in 1991 using modified Delphi method, consists in a list of 30 drugs to be avoided in nursing home residents regardless of diagnoses, dose and frequency of medication use. Updates reflected the appearance of new drugs and knowledge, and broadened application of the criteria to ambulatory elderly [13-15]. The latest version (Beers, 2002) considers 48 inappropriate medications or classes of medications regardless of diagnosis or conditions, and inappropriate medications or classes for 20 conditions. In 2001, Zhan et al. [19] classified 1997 Beers Criteria drugs into 3 categories: "always avoid", "rarely appropriate", and "some indications". In 2003, an expert panel classified the 2003 Beers Criteria drugs into the same three categories, but only the categories "always avoid" and "rarely appropriate" were included in the HEDIS criteria [30]. The McLeod method, which is also considered explicit, was developed by a Canadian panel of experts, and consists in 18 inappropriate medications for all elderly regardless of diagnoses or conditions, 16 inappropriate drug-disease interactions, and 4 inappropriate drug-drug interactions [20]. Some studies in this review use more than one criterion [23,24] or more than one version of the same criterion simultaneously [25].

Most of the studies adapt the explicit criteria to exclude items that depend on dosage, use frequency, diagnosis, or the drug's availability in the country of the study. These adaptations are explained in part by the use of administrative databases containing no details about the drugs or how they are used. Also, extrapolations are made to countries other than where the criterion originated, where dosages may not be the same and prescription habits may be different from the method's country of origin. Also observed were adaptations to include drugs with a pharmacological profile similar to those mentioned in the criterion and available in the study country. These facts indicate the difficulties involved in extrapolating criteria from the country of origin to others. These difficulties are reflected even in the choice of study population, as was observed in this review: most of the studies analyzed (79%) were conducted in the USA, the country of origin of the Beers method, which also predominated in IMU analysis (74% of the studies). In addition to the difficulties regarding interchangeability of criteria, the explicit methods are criticized for their lack of specificity, given that they do not consider the characteristics or clinical condition of each patient [11]. Accordingly, many authors prefer to include the term "potentially inappropriate" in their description of estimates.

In most of the studies that use multivariate analyses, IMU is associated with the female sex and advanced age (Table 2). Also in the multivariate analyses, the number of drugs used or prescribed seems to be the most important factor associated with IMU. In Table 2 the crude prevalence presented in single studies shows that polypharmacy is the covariate most strongly associated with IMU. This association suggests that the use of several drugs may also mean exposure to substances where the risks outweigh the benefits.

As regards the drugs/therapeutic classes most identified as inappropriate, the analysis of the medications was complicated by the heterogeneity of drug presentations. Classifications are not uniform and the rankings most used sometimes specify the drugs, sometimes the therapeutic classes, or even both drugs and therapeutic classes. Nonetheless, the studies do single out substances and therapeutic classes used for diseases highly prevalent among the world population, such as depression and anxiety [40]. The medications used to treat these diseases in the elderly are present in several explicit methods and associated with severe adverse events, such as sedation, falls and cognitive dysfunctions [13,41]. However, it is important to consider that only the short-acting benzodiazepines were strongly associated with fall-related injuries and that nowadays, the tricyclic antidepressants have been largely replaced by selective serotonin reuptake inhibitors because of lesser adverse effects [41]. Prescription of medication to treat these diseases thus deserves close attention, given that withdrawal of such medication is associated with a reduction in adverse effects, and improvements in physical and cognitive functions in the elderly [42].

This review was intended to contribute to knowledge about pharmacotherapy for the elderly by evaluating a non-institutionalized population. Our search strategy identified a large body of literature. Nonetheless, we may have missed relevant articles that were not identified, unpublished or excluded erroneously. Reliable evaluation of the vast and heterogeneous bibliographical material was assured by independent duplicate reading, and review by a third author at all stages of data selection and extraction. Certain limitations must be considered, however. Firstly, this review addressed only studies of administrative data sources, which are retrospective and have gaps in clinical information and in drug exposure data. On the other hand, they offer information on large populations. The number of articles published has been growing over the past few years. Contributions to the conceptual framework [43] and statistical approaches [44] have allowed a better understanding of the large administrative database as a valid means to examine the quality of medical services. Here, they were chosen for their representativeness, which yields more precise estimates and power to detect differences that otherwise would not offer statistical significance. As observed in Table 2 the confidence intervals of estimates for the association between inappropriate medication use and sex, age or number of medications are very small. Studies of administrative data sources may also be useful as inexpensive screening tools in areas where quality can be investigated in greater depth. Lastly, this review did not address the repercussions of inappropriate medication use on the health of the elderly nor the capacity of the methods used to predict adverse outcomes, both of which are important considerations for clinical practice.

As more studies are published, it may be possible to measure and record all potentially important covariates. These should be considered in future studies in order to improve the ability to identify their impact on the estimates and develop control strategies. Variables such as sex, age and total number of medications used should be mandatory in future studies. It is also important to give attention to other sources of information, such as medical records and surveys, with a view to ascertaining to what extent different study designs entail discrepant results. Moreover, in the future, reviews of articles that analyze primary data from population surveys - with information on social variables, demographics, health status, diseases, lifestyle habits, and physical and mental limitations - can enrich our understanding of the complex network of factors involved in prescribing drugs for the elderly.

## Conclusions

Inappropriate medication use is a public health problem and must be evaluated constantly as the panorama of pharmacotherapy changes. However, estimates of IMU can be influenced by diverse factors relating to the detection method used and the study population.

Identifying vulnerable patient groups and developing pharmacological alternatives suited to country-specific conditions are important strategies for orienting clinical conduct and risk reduction in this age group.

From reading the articles, the authors identified certain salient problem areas, which could be worked around in the future. Prominent among them is the applicability of the list of drugs. There is a need for scientific evidence-based lists to be drawn up with clearly defined indicators of inappropriate medication prescription, as well as drug-drug and drug-disease interactions [45]. This recommendation is even more important in the case of large administrative data bases. This would then provide an easily applicable tool with major potential for research and monitoring, to be used by researchers and health system managers. Another still unsolved problem relates to the existence of lists compiled by only a few countries. It is important to develop lists appropriate to the products on sale in each country, so as to make it easier to operationalize studies, and for surveillance systems to monitor. In addition, the inclusion of lists of medications inappropriate for the elderly on national drug formularies would reduce their prescription and use in this age group [46]. However, the development of more suitable criteria of inappropriateness does not itself guarantee reduced prevalence of IMU. Efforts to identify factors associated with IMU may help policy makers identify vulnerable patient groups and develop programs to modify prescription patterns [47,48]. Studies of large administrative data bases, such as those analyzed in this study, can make a major contribution in this respect. However, it is essential to develop effective approaches. Geriatric medicine services, pharmacist interventions in patient care, implementation of appropriate prescription criteria and computerized decision-making support systems can improve the appropriateness of prescribing for the elderly in ambulatory-care settings [49-52]. The review by Forsetlund et al [53] shows that, in nursing homes, under certain circumstances, interventions using educational outreach, on-site education alone or as part of an intervention package and pharmacists medication review may reduce inappropriate medication drug use.

## Competing interests

The authors declare that they have no competing interests.

## Authors' contributions

LG, FGC and SR were involved in the conceptualization of the research question. LG, FGC, GSD and SR devised the search strategy, and identified and appraised relevant literature. SR acted as third reviewer, assisting in appraisal and interpretation of relevant studies where agreement could not be met. LG drafted the manuscript with critical input from all other authors. All authors read and approved the final manuscript.

## Pre-publication history

The pre-publication history for this paper can be accessed here:

http://www.biomedcentral.com/1471-2318/11/79/prepub

## Supplementary Material

Additional file 1 **Search Strategy**. the file presents the complete search strategy performed in Medline and Embase databases. Click here for file
