# Supplementary material for: Inappropriate medication use among the elderly: a systematic review of administrative databases
Source: BMC Geriatr. 2011 Nov 30;11:79. doi: 10.1186/1471-2318-11-79 (PMC3267683; doi:10.1186/1471-2318-11-79)
Supplement: Additional file 1 — Search Strategy. the file presents the complete search strategy performed in Medline and Embase databases. [file 1471-2318-11-79-S1.DOC]

**Search Strategy**

Database: Medline

| **ID** | **Search** |
| --- | --- |
| #1 | "aged"[MeSH Terms] |
| #2 | "frail elderly"[MeSH Terms] |
| #3 | #1 NOT #2 |
| #4 | (("drug therapy"[MeSH Terms]) OR ((drug utilization[MeSH Terms]) OR ("pharmaceutical preparations"[MeSH Terms]) OR ("drug interactions"[MeSH Terms])) |
| #5 | (("inappropriate drug"[Title/Abstract]) OR ("inappropriate drugs"[Title/Abstract]) OR ("inappropriate medication"[Title/Abstract]) OR ("inappropriate medications"[Title/Abstract]) OR ("inappropriate medicines"[Title/Abstract] )OR ("inappropriate prescribing"[Title/Abstract] )OR ("inappropriate prescription"[Title/Abstract]) OR ("inappropriate prescriptions"[Title/Abstract]) OR ("inadequate medication"[Title/Abstract]) OR ("suboptimal therapy"[Title/Abstract]) OR ("suboptimal prescribing"[Title/Abstract])) |
| #6 | #4 OR #5 |
| #7 | #3 AND #6 |
| #8 | #7 Limits: **Publication Date from 1990/01 to 2010/06, Humans** |
| #9 | #8 Limits: **Publication Date from 1990/01 to 2010/06, Humans, Clinical Trial, Editorial, Letter, Practice Guideline, Review** |
| #10 | #8 NOT #9 |

Database: Embase

| **ID** | **Search** |
| --- | --- |
| #1 | " inappropriate " |
| #2 | "geriatric patient'/exp OR 'aging'/exp OR 'aged'/exp" |
| #3 | “prescription'/exp OR 'prescription drug'/exp OR 'inappropriate prescribing'/exp” |
| #4 | #1 AND #2 AND #3 AND ([article]/lim OR [article in press]/lim OR [conference abstract]/lim OR [conference paper]/lim OR [conference review]/lim OR [erratum]/lim OR [short survey]/lim) AND [humans]/lim AND [1990-2010]/py” |
